# 1-Benzyl-6,8-dimethyl-4-phenyl-2-tosyl-1,2,3,3a,4,6,7,8,9,9b-deca­hydro­pyrrolo[3,4-*c*]pyrano[6,5-*d*]pyrimidine-7,9-dione

**DOI:** 10.1107/S1600536809044341

**Published:** 2009-10-28

**Authors:** K. Chinnakali, D. Sudha, M. Jayagobi, R. Raghunathan, Hoong-Kun Fun

**Affiliations:** a Department of Physics, Anna University Chennai, Chennai 600 025, India; b Department of Organic Chemistry, University of Madras, Guindy Campus, Chennai 600 025, India; c X-ray Crystallography Unit, School of Physics, Universiti Sains Malaysia, 11800 USM, Penang, Malaysia

## Abstract

The mol­ecule of the title compound, C_31_H_31_N_3_O_5_S, adopts a folded conformation, with the sulfonyl-bound phenyl ring lying over the pyrimidine ring [dihedral angle = 12.04 (6)° and centroid–centroid separation = 3.6986 (8) Å]. The pyrrolidine ring adopts a twist conformation, the dihydro­pyran ring is in a half-chair conformation and the two rings are *cis*-fused. The tosyl group is attached to the pyrrolidine ring in an equatorial position while the benzyl group is axially attached. The mol­ecular structure is stabilized by weak C—H⋯O hydrogen bonds and C—H⋯π inter­actions. In the crystal, pairs of mol­ecules related by inversion symmetry are linked by C—H⋯O hydrogen bonds, forming chains propagating along the *c* axis which are cross-linked into a three-dimensional framework by further C—H⋯O links.

## Related literature

For the biological activity of pyran­opyrimidine derivatives, see: Abdel Fattah *et al.* (2004 ▶); Bedair *et al.* (2000 ▶, 2001 ▶); Bruno *et al.* (2000 ▶); Eid *et al.* (2004 ▶); Shamroukh *et al.* (2007 ▶). For a related structure, see: Chinnakali *et al.* (2007 ▶). For ring puckering parameters, see: Cremer & Pople (1975 ▶) and for asymmetry parameters, see: Duax *et al.* (1976 ▶). For hydrogen-bond motifs, see: Bernstein *et al.* (1995 ▶).
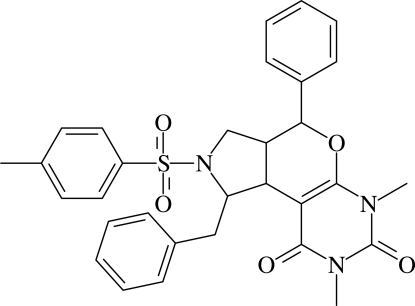


## Experimental

### 

#### Crystal data

C_31_H_31_N_3_O_5_S
*M* *_r_* = 557.65
Monoclinic, 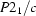

*a* = 13.2778 (2) Å
*b* = 15.8888 (2) Å
*c* = 13.0886 (2) Å
β = 99.019 (1)°
*V* = 2727.14 (7) Å^3^
*Z* = 4
Mo *K*α radiation
μ = 0.17 mm^−1^
*T* = 100 K
0.29 × 0.24 × 0.15 mm

#### Data collection

Bruker SMART APEXII CCD diffractometer
Absorption correction: multi-scan (*SADABS*; Bruker, 2005 ▶) *T*_min_ = 0.875, *T*_max_ = 0.976
39155 measured reflections
7888 independent reflections
6269 reflections with *I* > 2σ(*I*)
*R*_int_ = 0.042

#### Refinement

*R*[*F* ^2^ > 2σ(*F* ^2^)] = 0.043
*wR*(*F* ^2^) = 0.127
*S* = 1.07
7888 reflections
363 parameters
H-atom parameters constrained
Δρ_max_ = 0.38 e Å^−3^
Δρ_min_ = −0.45 e Å^−3^

### 

Data collection: *APEX2* (Bruker, 2005 ▶); cell refinement: *SAINT* (Bruker, 2005 ▶); data reduction: *SAINT*; program(s) used to solve structure: *SHELXTL* (Sheldrick, 2008 ▶); program(s) used to refine structure: *SHELXTL*; molecular graphics: *SHELXTL*; software used to prepare material for publication: *SHELXTL* and *PLATON* (Spek, 2009 ▶).

## Supplementary Material

Crystal structure: contains datablocks global, I. DOI: 10.1107/S1600536809044341/hb5182sup1.cif

Structure factors: contains datablocks I. DOI: 10.1107/S1600536809044341/hb5182Isup2.hkl

Additional supplementary materials:  crystallographic information; 3D view; checkCIF report

## Figures and Tables

**Table 1:** Hydrogen-bond geometry (Å, °)

*D*—H⋯*A*	*D*—H	H⋯*A*	*D*⋯*A*	*D*—H⋯*A*
C4—H4⋯O5	0.98	2.45	3.0586 (16)	120
C2—H2⋯*Cg*1	0.98	2.67	3.5377 (15)	148
C22—H22⋯O4^i^	0.93	2.57	3.258 (2)	132
C24—H24*B*⋯O5^ii^	0.96	2.48	3.4236 (17)	169
C30—H30⋯O1^iii^	0.93	2.40	3.180 (2)	142

Symmetry codes: (i) 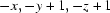; (ii) 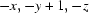; (iii) 
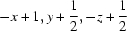
. *Cg*1 is the centroid of the C26–C31 ring.

**Fig. 1.**
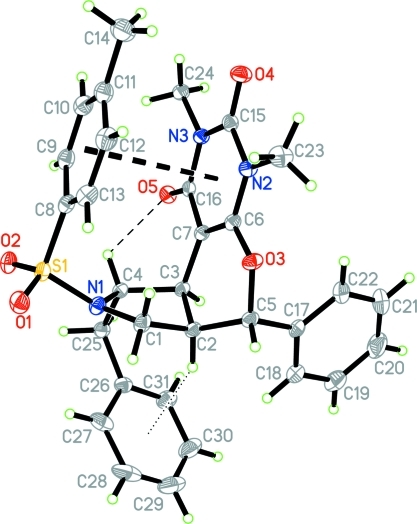
The molecular structure of (I) with displacement ellipsoids drawn at the 50% probability level. Dashed and dotted lines indicate C—H···O and C—H···π interactions, respectively. The π-π interaction is shown by a dashed solid line.

**Fig. 2.**
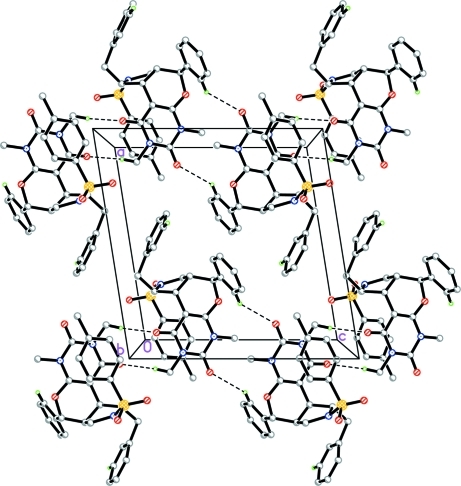
Part of the crystal packing of (I), showing hydrogen-bonded (dashed lines) chains. For the sake of clarity, H atoms not involved in intermolecular hydrogen bonds have been omitted.

## supplementary crystallographic information

### Comment

Pyranopyrimidine derivatives exhibit antipyretic, analgesic and antiplatelet (Bruno *et al.*, 2000), antiviral (Shamroukh *et al.*, 2007) and antimicrobial (Bedair *et al.*, 2000, 2001; Eid *et al.*, 2004; Abdel Fattah *et al.*, 2004) activities. We report here the crystal structure of the title compound, a pyranopyrimidine derivative.

Bond lengths and angles are comparable with those observed in a related structure (Chinnakali *et al.*, 2007). The pyrimidine ring is planar, with an r.m.s. deviation of fitted atoms of 0.032 Å, and atoms O4, O5, C23 and C24 deviating by 0.079 (1), -0.146 (1), 0.021 (2) and 0.142 (2) Å, respectively. The pyrrolidine ring adopts a twist conformation, with twist about the C2—C3 bond; the puckering parameters (Cremer & Pople, 1975) q2 = 0.345 (2) Å and φ = 87.2 (2)°, and asymmetry parameter (Duax *et al.*, 1976) ΔC_2_[C2—C3] = 3.13 (14)°. The tosyl group is attached to the pyrrolidine ring in an equatorial position while the benzyl group is axially attached. The dihydropyran ring adopts a half-chair conformation, with atoms C2 and C5 deviating from the O3/C3/C6/C7 plane by -0.302 (2) Å and 0.427 (2) Å, respectively. The asymmetry parameter ΔC_2_[C2—C5] is 6.4 (2)°, and the puckering parameters Q, θ and φ are 0.478 (1) Å, 54.1 (2)° and 86.1 (2)°, respectively. The phenyl group is axially attached to the dihydropyran ring. The pyrrolidine ring is *cis*-fused to the dihydropyran ring.

The molecule is in a folded conformation, with the sulfonyl-bound phenyl ring lying over the pyrimidine ring [dihedral angle = 12.04 (6)°]. The folded conformation is stabilized by weak π-π interaction between phenyl and pyrimidine rings (centroid-to-centroid distance = 3.6986 (8) Å), C4—H4···O5 hydrogen bonds and C2—H2···π interaction involving the benzyl phenyl ring (C26—C31). The dihedral angle between the C17—C22 and C26—C31 phenyl rings is 57.50 (8)°.

Pairs of molecules related by inversion are linked into *R*_2_^2^(10) (Bernstein *et al.*, 1995) dimers by C24—H24B···O5 hydrogen bonds, and dimers related by inversion are linked into chains along the *c* axis by pairs of C22—H22···O4 hydrogen bonds which generate an *R*_2_^2^(18) motif. The adjacent chains are cross-linked through C30—H30···O1 hydrogen bonds to form a three-dimensional framework.

### Experimental

To a solution of 1,3-dimethyl-pyrimidine-2,4,6-trione (1 mmol) in dry toluene (20 ml), the corresponding 2-(*N*-cinnamyl-*N*-tosylamino)acetaldehyde (1 mmol) and catalytic amount of the base ethylenediamine-*N*,*N*'-diacetate (EDDA) were added and the reaction mixture was refluxed for 12 h. After completion of reaction, the solvent was evaporated under reduced pressure and the crude product was chromatographed using a hexane-ethyl acetate (8:2 *v*/*v*) mixture to obtain the title compound. The compound was recrystallized from ethyl acetate solution by slow evaporation to yield colourless blocks of (I).

### Refinement

H atoms were positioned geometrically and allowed to ride on their parent atoms, with C—H = 0.93–0.98 Å and *U*_iso_(H) = 1.2–1.5(methyl) *U*_eq_(C). The H atoms of the tosyl methyl group are disordered over two orientations and two sets of idealized positions rotated from each other by 60° were used. A rotating group model was used for the normal methyl groups.

### Crystal data

**Table:**

C_31_H_31_N_3_O_5_S	*F*(000) = 1176
*M**_r_* = 557.65	*D*_x_ = 1.358 Mg m^−^^3^
Monoclinic, *P*2_1_/*c*	Mo *K*α radiation, λ = 0.71073 Å
Hall symbol: -P 2ybc	Cell parameters from 9929 reflections
*a* = 13.2778 (2) Å	θ = 2.6–32.3°
*b* = 15.8888 (2) Å	µ = 0.17 mm^−^^1^
*c* = 13.0886 (2) Å	*T* = 100 K
β = 99.019 (1)°	Block, colourless
*V* = 2727.14 (7) Å^3^	0.29 × 0.24 × 0.15 mm
*Z* = 4	

### Data collection

**Table:**

Bruker SMART APEXII CCD diffractometer	7888 independent reflections
Radiation source: fine-focus sealed tube	6269 reflections with *I* > 2σ(*I*)
graphite	*R*_int_ = 0.042
φ and ω scans	θ_max_ = 30.0°, θ_min_ = 2.0°
Absorption correction: multi-scan (*SADABS*; Bruker, 2005)	*h* = −17→18
*T*_min_ = 0.875, *T*_max_ = 0.976	*k* = −22→21
39155 measured reflections	*l* = −17→18

### Refinement

**Table:**

Refinement on *F*^2^	Primary atom site location: structure-invariant direct methods
Least-squares matrix: full	Secondary atom site location: difference Fourier map
*R*[*F*^2^ > 2σ(*F*^2^)] = 0.043	Hydrogen site location: inferred from neighbouring sites
*wR*(*F*^2^) = 0.127	H-atom parameters constrained
*S* = 1.07	*w* = 1/[σ^2^(*F*_o_^2^) + (0.0645*P*)^2^ + 0.8585*P*] where *P* = (*F*_o_^2^ + 2*F*_c_^2^)/3
7888 reflections	(Δ/σ)_max_ = 0.001
363 parameters	Δρ_max_ = 0.38 e Å^−^^3^
0 restraints	Δρ_min_ = −0.45 e Å^−^^3^

### Special details

**Table:**

Geometry. All e.s.d.'s (except the e.s.d. in the dihedral angle between two l.s. planes) are estimated using the full covariance matrix. The cell e.s.d.'s are taken into account individually in the estimation of e.s.d.'s in distances, angles and torsion angles; correlations between e.s.d.'s in cell parameters are only used when they are defined by crystal symmetry. An approximate (isotropic) treatment of cell e.s.d.'s is used for estimating e.s.d.'s involving l.s. planes.
Refinement. Refinement of *F*^2^ against ALL reflections. The weighted *R*-factor *wR* and goodness of fit *S* are based on *F*^2^, conventional *R*-factors *R* are based on *F*, with *F* set to zero for negative *F*^2^. The threshold expression of *F*^2^ > σ(*F*^2^) is used only for calculating *R*-factors(gt) *etc*. and is not relevant to the choice of reflections for refinement. *R*-factors based on *F*^2^ are statistically about twice as large as those based on *F*, and *R*- factors based on ALL data will be even larger.

### Fractional atomic coordinates and isotropic or equivalent isotropic displacement parameters (Å^2^)

**Table:**

	*x*	*y*	*z*	*U*_iso_*/*U*_eq_	Occ. (<1)
S1	0.24002 (3)	0.27919 (2)	0.08504 (3)	0.01786 (9)	
O1	0.29691 (8)	0.20418 (7)	0.11604 (8)	0.0244 (2)	
O2	0.22163 (8)	0.30362 (7)	−0.02164 (8)	0.0227 (2)	
O3	0.22116 (7)	0.42241 (7)	0.41969 (7)	0.0192 (2)	
O4	−0.12019 (8)	0.44236 (7)	0.32394 (8)	0.0230 (2)	
O5	0.07992 (7)	0.55421 (6)	0.10506 (7)	0.0180 (2)	
N1	0.30062 (9)	0.35642 (7)	0.14908 (9)	0.0172 (2)	
N2	0.05214 (9)	0.43750 (7)	0.37124 (9)	0.0169 (2)	
N3	−0.02062 (8)	0.49601 (7)	0.21216 (8)	0.0154 (2)	
C1	0.33158 (11)	0.34751 (9)	0.26213 (10)	0.0193 (3)	
H1A	0.3954	0.3171	0.2781	0.023*	
H1B	0.2797	0.3184	0.2931	0.023*	
C2	0.34345 (10)	0.43876 (9)	0.30011 (10)	0.0161 (3)	
H2	0.4127	0.4579	0.2954	0.019*	
C3	0.26717 (10)	0.48911 (8)	0.22261 (10)	0.0142 (2)	
H3	0.2889	0.5479	0.2195	0.017*	
C4	0.27054 (10)	0.44416 (8)	0.11846 (10)	0.0151 (2)	
H4	0.2019	0.4438	0.0779	0.018*	
C5	0.32387 (10)	0.45051 (9)	0.41091 (10)	0.0179 (3)	
H5	0.3721	0.4147	0.4558	0.021*	
C6	0.14751 (10)	0.44924 (8)	0.34517 (10)	0.0155 (2)	
C7	0.16259 (10)	0.48378 (8)	0.25380 (10)	0.0145 (2)	
C8	0.12079 (11)	0.26992 (9)	0.12787 (11)	0.0182 (3)	
C9	0.03512 (11)	0.31028 (9)	0.07404 (11)	0.0203 (3)	
H9	0.0408	0.3436	0.0168	0.024*	
C10	−0.05862 (12)	0.30012 (9)	0.10679 (12)	0.0233 (3)	
H10	−0.1159	0.3261	0.0701	0.028*	
C11	−0.06866 (12)	0.25174 (9)	0.19372 (12)	0.0241 (3)	
C12	0.01881 (12)	0.21432 (9)	0.24816 (12)	0.0247 (3)	
H12	0.0139	0.1836	0.3077	0.030*	
C13	0.11282 (12)	0.22190 (9)	0.21553 (11)	0.0220 (3)	
H13	0.1699	0.1953	0.2517	0.026*	
C14	−0.17063 (13)	0.23950 (12)	0.22861 (15)	0.0331 (4)	
H14A	−0.2101	0.2902	0.2164	0.050*	0.50
H14B	−0.1605	0.2266	0.3011	0.050*	0.50
H14C	−0.2063	0.1940	0.1905	0.050*	0.50
H14D	−0.1745	0.1836	0.2557	0.050*	0.50
H14E	−0.2241	0.2472	0.1709	0.050*	0.50
H14F	−0.1782	0.2798	0.2815	0.050*	0.50
C15	−0.03560 (10)	0.45740 (9)	0.30321 (10)	0.0168 (3)	
C16	0.07514 (10)	0.51458 (8)	0.18497 (10)	0.0141 (2)	
C17	0.34013 (11)	0.54080 (9)	0.44869 (10)	0.0192 (3)	
C18	0.42511 (12)	0.58588 (10)	0.42851 (12)	0.0237 (3)	
H18	0.4716	0.5604	0.3921	0.028*	
C19	0.44095 (13)	0.66829 (11)	0.46223 (14)	0.0316 (4)	
H19	0.4976	0.6979	0.4480	0.038*	
C20	0.37218 (15)	0.70650 (12)	0.51717 (15)	0.0389 (4)	
H20	0.3824	0.7619	0.5395	0.047*	
C21	0.28856 (15)	0.66214 (13)	0.53870 (14)	0.0388 (4)	
H21	0.2426	0.6878	0.5757	0.047*	
C22	0.27261 (13)	0.57937 (11)	0.50547 (12)	0.0282 (3)	
H22	0.2167	0.5497	0.5212	0.034*	
C23	0.03991 (12)	0.39397 (11)	0.46792 (11)	0.0257 (3)	
H23A	0.0907	0.4137	0.5231	0.039*	
H23B	0.0477	0.3344	0.4593	0.039*	
H23C	−0.0267	0.4054	0.4844	0.039*	
C24	−0.11133 (10)	0.51453 (10)	0.13584 (11)	0.0194 (3)	
H24A	−0.1674	0.4807	0.1501	0.029*	
H24B	−0.0974	0.5020	0.0677	0.029*	
H24C	−0.1285	0.5730	0.1398	0.029*	
C25	0.34547 (10)	0.48309 (9)	0.05318 (10)	0.0173 (3)	
H25A	0.3490	0.4471	−0.0060	0.021*	
H25B	0.3193	0.5373	0.0274	0.021*	
C26	0.45176 (10)	0.49478 (10)	0.11237 (11)	0.0195 (3)	
C27	0.51924 (12)	0.42716 (11)	0.13215 (13)	0.0274 (3)	
H27	0.5021	0.3748	0.1027	0.033*	
C28	0.61285 (13)	0.43864 (14)	0.19648 (14)	0.0385 (5)	
H28	0.6568	0.3931	0.2109	0.046*	
C29	0.64105 (13)	0.51625 (15)	0.23891 (13)	0.0411 (5)	
H29	0.7032	0.5228	0.2821	0.049*	
C30	0.57666 (13)	0.58375 (14)	0.21684 (13)	0.0364 (4)	
H30	0.5959	0.6366	0.2435	0.044*	
C31	0.48241 (12)	0.57272 (11)	0.15439 (12)	0.0254 (3)	
H31	0.4390	0.6186	0.1405	0.030*	

### Atomic displacement parameters (Å^2^)

**Table:**

	*U*^11^	*U*^22^	*U*^33^	*U*^12^	*U*^13^	*U*^23^
S1	0.02082 (17)	0.01614 (16)	0.01588 (16)	0.00094 (12)	0.00059 (12)	−0.00164 (12)
O1	0.0276 (6)	0.0181 (5)	0.0265 (5)	0.0056 (4)	0.0010 (4)	−0.0017 (4)
O2	0.0285 (5)	0.0242 (5)	0.0148 (5)	−0.0001 (4)	0.0019 (4)	−0.0030 (4)
O3	0.0159 (5)	0.0257 (5)	0.0149 (4)	−0.0012 (4)	−0.0014 (3)	0.0062 (4)
O4	0.0166 (5)	0.0317 (6)	0.0213 (5)	−0.0017 (4)	0.0048 (4)	0.0035 (4)
O5	0.0201 (5)	0.0194 (5)	0.0143 (4)	0.0030 (4)	0.0025 (4)	0.0036 (4)
N1	0.0193 (6)	0.0157 (5)	0.0155 (5)	0.0001 (4)	−0.0008 (4)	0.0001 (4)
N2	0.0173 (5)	0.0196 (6)	0.0137 (5)	−0.0011 (4)	0.0018 (4)	0.0049 (4)
N3	0.0137 (5)	0.0182 (5)	0.0137 (5)	0.0014 (4)	0.0002 (4)	0.0012 (4)
C1	0.0216 (7)	0.0191 (7)	0.0156 (6)	0.0030 (5)	−0.0024 (5)	0.0004 (5)
C2	0.0149 (6)	0.0175 (6)	0.0147 (6)	0.0003 (5)	−0.0011 (5)	−0.0008 (5)
C3	0.0136 (6)	0.0158 (6)	0.0126 (5)	−0.0003 (5)	0.0001 (4)	0.0014 (4)
C4	0.0150 (6)	0.0163 (6)	0.0134 (6)	−0.0001 (5)	0.0001 (4)	0.0005 (5)
C5	0.0152 (6)	0.0237 (7)	0.0138 (6)	0.0000 (5)	−0.0008 (5)	0.0017 (5)
C6	0.0157 (6)	0.0149 (6)	0.0150 (6)	0.0002 (5)	−0.0006 (5)	0.0008 (5)
C7	0.0151 (6)	0.0146 (6)	0.0132 (6)	0.0000 (5)	0.0002 (4)	0.0005 (4)
C8	0.0210 (7)	0.0141 (6)	0.0183 (6)	−0.0017 (5)	−0.0003 (5)	−0.0019 (5)
C9	0.0252 (7)	0.0159 (6)	0.0185 (6)	0.0005 (5)	−0.0004 (5)	−0.0008 (5)
C10	0.0230 (7)	0.0178 (7)	0.0274 (7)	0.0027 (5)	−0.0013 (6)	−0.0029 (6)
C11	0.0260 (7)	0.0169 (7)	0.0297 (7)	−0.0018 (6)	0.0056 (6)	−0.0053 (6)
C12	0.0308 (8)	0.0196 (7)	0.0238 (7)	−0.0039 (6)	0.0041 (6)	0.0014 (5)
C13	0.0242 (7)	0.0176 (7)	0.0225 (7)	−0.0013 (5)	−0.0010 (5)	0.0031 (5)
C14	0.0290 (8)	0.0312 (9)	0.0410 (9)	−0.0012 (7)	0.0117 (7)	−0.0034 (7)
C15	0.0178 (6)	0.0176 (6)	0.0147 (6)	0.0010 (5)	0.0021 (5)	0.0004 (5)
C16	0.0160 (6)	0.0135 (6)	0.0126 (5)	0.0012 (5)	0.0010 (4)	−0.0011 (4)
C17	0.0200 (7)	0.0240 (7)	0.0120 (6)	0.0001 (5)	−0.0022 (5)	−0.0008 (5)
C18	0.0213 (7)	0.0272 (8)	0.0215 (7)	−0.0011 (6)	−0.0001 (5)	−0.0031 (6)
C19	0.0292 (8)	0.0299 (9)	0.0335 (9)	−0.0066 (7)	−0.0023 (7)	−0.0040 (7)
C20	0.0445 (10)	0.0314 (9)	0.0379 (10)	−0.0029 (8)	−0.0024 (8)	−0.0151 (8)
C21	0.0434 (10)	0.0434 (11)	0.0306 (9)	0.0022 (8)	0.0091 (8)	−0.0166 (8)
C22	0.0299 (8)	0.0360 (9)	0.0195 (7)	−0.0016 (7)	0.0061 (6)	−0.0063 (6)
C23	0.0256 (7)	0.0331 (8)	0.0189 (7)	−0.0027 (6)	0.0050 (5)	0.0124 (6)
C24	0.0149 (6)	0.0253 (7)	0.0166 (6)	0.0025 (5)	−0.0014 (5)	0.0002 (5)
C25	0.0166 (6)	0.0207 (7)	0.0145 (6)	0.0000 (5)	0.0020 (5)	0.0012 (5)
C26	0.0163 (6)	0.0268 (7)	0.0160 (6)	−0.0005 (5)	0.0043 (5)	0.0034 (5)
C27	0.0222 (7)	0.0347 (9)	0.0277 (8)	0.0071 (6)	0.0108 (6)	0.0083 (6)
C28	0.0204 (8)	0.0622 (13)	0.0346 (9)	0.0161 (8)	0.0095 (7)	0.0195 (9)
C29	0.0188 (8)	0.0798 (15)	0.0239 (8)	−0.0074 (9)	0.0011 (6)	0.0083 (9)
C30	0.0258 (8)	0.0571 (12)	0.0256 (8)	−0.0166 (8)	0.0023 (6)	−0.0016 (8)
C31	0.0227 (7)	0.0303 (8)	0.0228 (7)	−0.0057 (6)	0.0020 (6)	0.0032 (6)

### Geometric parameters (Å, °)

**Table:**

S1—O2	1.4328 (10)	C12—H12	0.93
S1—O1	1.4351 (11)	C13—H13	0.93
S1—N1	1.6261 (12)	C14—H14A	0.96
S1—C8	1.7664 (15)	C14—H14B	0.96
O3—C6	1.3380 (15)	C14—H14C	0.96
O3—C5	1.4566 (16)	C14—H14D	0.96
O4—C15	1.2198 (17)	C14—H14E	0.96
O5—C16	1.2309 (15)	C14—H14F	0.96
N1—C1	1.4791 (17)	C17—C22	1.393 (2)
N1—C4	1.4878 (17)	C17—C18	1.396 (2)
N2—C6	1.3748 (18)	C18—C19	1.387 (2)
N2—C15	1.3873 (17)	C18—H18	0.93
N2—C23	1.4733 (17)	C19—C20	1.388 (3)
N3—C15	1.3822 (17)	C19—H19	0.93
N3—C16	1.4043 (17)	C20—C21	1.381 (3)
N3—C24	1.4690 (16)	C20—H20	0.93
C1—C2	1.5327 (19)	C21—C22	1.391 (3)
C1—H1A	0.97	C21—H21	0.93
C1—H1B	0.97	C22—H22	0.93
C2—C5	1.5245 (18)	C23—H23A	0.96
C2—C3	1.5403 (17)	C23—H23B	0.96
C2—H2	0.98	C23—H23C	0.96
C3—C7	1.5100 (18)	C24—H24A	0.96
C3—C4	1.5457 (18)	C24—H24B	0.96
C3—H3	0.98	C24—H24C	0.96
C4—C25	1.5391 (19)	C25—C26	1.5111 (19)
C4—H4	0.98	C25—H25A	0.97
C5—C17	1.522 (2)	C25—H25B	0.97
C5—H5	0.98	C26—C31	1.390 (2)
C6—C7	1.3589 (18)	C26—C27	1.397 (2)
C7—C16	1.4392 (17)	C27—C28	1.400 (2)
C8—C13	1.396 (2)	C27—H27	0.93
C8—C9	1.3965 (19)	C28—C29	1.380 (3)
C9—C10	1.388 (2)	C28—H28	0.93
C9—H9	0.93	C29—C30	1.374 (3)
C10—C11	1.397 (2)	C29—H29	0.93
C10—H10	0.93	C30—C31	1.394 (2)
C11—C12	1.397 (2)	C30—H30	0.93
C11—C14	1.508 (2)	C31—H31	0.93
C12—C13	1.386 (2)		
			
O2—S1—O1	120.15 (7)	C11—C14—H14D	109.5
O2—S1—N1	107.03 (6)	H14A—C14—H14D	141.1
O1—S1—N1	106.55 (6)	H14B—C14—H14D	56.3
O2—S1—C8	107.91 (6)	H14C—C14—H14D	56.3
O1—S1—C8	107.41 (7)	C11—C14—H14E	109.5
N1—S1—C8	107.15 (6)	H14A—C14—H14E	56.3
C6—O3—C5	115.54 (10)	H14B—C14—H14E	141.1
C1—N1—C4	112.33 (10)	H14C—C14—H14E	56.3
C1—N1—S1	118.75 (9)	H14D—C14—H14E	109.5
C4—N1—S1	118.55 (8)	C11—C14—H14F	109.5
C6—N2—C15	121.56 (11)	H14A—C14—H14F	56.3
C6—N2—C23	120.44 (11)	H14B—C14—H14F	56.3
C15—N2—C23	117.62 (12)	H14C—C14—H14F	141.1
C15—N3—C16	124.76 (11)	H14D—C14—H14F	109.5
C15—N3—C24	117.50 (11)	H14E—C14—H14F	109.5
C16—N3—C24	117.64 (11)	O4—C15—N3	122.73 (12)
N1—C1—C2	103.43 (11)	O4—C15—N2	121.50 (12)
N1—C1—H1A	111.1	N3—C15—N2	115.76 (12)
C2—C1—H1A	111.1	O5—C16—N3	119.48 (11)
N1—C1—H1B	111.1	O5—C16—C7	124.18 (12)
C2—C1—H1B	111.1	N3—C16—C7	116.33 (11)
H1A—C1—H1B	109.0	C22—C17—C18	118.82 (14)
C5—C2—C1	113.59 (12)	C22—C17—C5	121.18 (14)
C5—C2—C3	111.73 (11)	C18—C17—C5	119.99 (13)
C1—C2—C3	104.73 (10)	C19—C18—C17	120.71 (15)
C5—C2—H2	108.9	C19—C18—H18	119.6
C1—C2—H2	108.9	C17—C18—H18	119.6
C3—C2—H2	108.9	C18—C19—C20	119.91 (17)
C7—C3—C2	109.29 (11)	C18—C19—H19	120.0
C7—C3—C4	111.56 (10)	C20—C19—H19	120.0
C2—C3—C4	103.52 (10)	C21—C20—C19	119.86 (17)
C7—C3—H3	110.7	C21—C20—H20	120.1
C2—C3—H3	110.7	C19—C20—H20	120.1
C4—C3—H3	110.7	C20—C21—C22	120.44 (17)
N1—C4—C25	110.89 (11)	C20—C21—H21	119.8
N1—C4—C3	103.83 (10)	C22—C21—H21	119.8
C25—C4—C3	114.49 (11)	C21—C22—C17	120.24 (16)
N1—C4—H4	109.1	C21—C22—H22	119.9
C25—C4—H4	109.1	C17—C22—H22	119.9
C3—C4—H4	109.1	N2—C23—H23A	109.5
O3—C5—C17	110.50 (11)	N2—C23—H23B	109.5
O3—C5—C2	109.81 (10)	H23A—C23—H23B	109.5
C17—C5—C2	112.83 (11)	N2—C23—H23C	109.5
O3—C5—H5	107.8	H23A—C23—H23C	109.5
C17—C5—H5	107.8	H23B—C23—H23C	109.5
C2—C5—H5	107.8	N3—C24—H24A	109.5
O3—C6—C7	125.35 (12)	N3—C24—H24B	109.5
O3—C6—N2	111.86 (11)	H24A—C24—H24B	109.5
C7—C6—N2	122.79 (12)	N3—C24—H24C	109.5
C6—C7—C16	118.20 (12)	H24A—C24—H24C	109.5
C6—C7—C3	121.95 (11)	H24B—C24—H24C	109.5
C16—C7—C3	119.84 (11)	C26—C25—C4	113.45 (11)
C13—C8—C9	120.33 (14)	C26—C25—H25A	108.9
C13—C8—S1	119.58 (11)	C4—C25—H25A	108.9
C9—C8—S1	120.08 (11)	C26—C25—H25B	108.9
C10—C9—C8	119.33 (14)	C4—C25—H25B	108.9
C10—C9—H9	120.3	H25A—C25—H25B	107.7
C8—C9—H9	120.3	C31—C26—C27	118.19 (14)
C9—C10—C11	121.35 (14)	C31—C26—C25	120.30 (13)
C9—C10—H10	119.3	C27—C26—C25	121.39 (14)
C11—C10—H10	119.3	C26—C27—C28	119.64 (17)
C10—C11—C12	118.17 (15)	C26—C27—H27	120.2
C10—C11—C14	121.51 (15)	C28—C27—H27	120.2
C12—C11—C14	120.33 (15)	C29—C28—C27	121.20 (17)
C13—C12—C11	121.51 (14)	C29—C28—H28	119.4
C13—C12—H12	119.2	C27—C28—H28	119.4
C11—C12—H12	119.2	C30—C29—C28	119.50 (16)
C12—C13—C8	119.26 (14)	C30—C29—H29	120.3
C12—C13—H13	120.4	C28—C29—H29	120.3
C8—C13—H13	120.4	C29—C30—C31	119.80 (18)
C11—C14—H14A	109.5	C29—C30—H30	120.1
C11—C14—H14B	109.5	C31—C30—H30	120.1
H14A—C14—H14B	109.5	C26—C31—C30	121.62 (17)
C11—C14—H14C	109.5	C26—C31—H31	119.2
H14A—C14—H14C	109.5	C30—C31—H31	119.2
H14B—C14—H14C	109.5		
			
O2—S1—N1—C1	−178.78 (10)	S1—C8—C9—C10	177.94 (11)
O1—S1—N1—C1	−49.05 (12)	C8—C9—C10—C11	1.3 (2)
C8—S1—N1—C1	65.68 (11)	C9—C10—C11—C12	0.8 (2)
O2—S1—N1—C4	38.87 (12)	C9—C10—C11—C14	−179.07 (14)
O1—S1—N1—C4	168.59 (10)	C10—C11—C12—C13	−2.3 (2)
C8—S1—N1—C4	−76.68 (11)	C14—C11—C12—C13	177.51 (14)
C4—N1—C1—C2	−12.74 (15)	C11—C12—C13—C8	1.8 (2)
S1—N1—C1—C2	−157.29 (9)	C9—C8—C13—C12	0.4 (2)
N1—C1—C2—C5	151.72 (11)	S1—C8—C13—C12	−179.44 (11)
N1—C1—C2—C3	29.54 (14)	C16—N3—C15—O4	179.99 (13)
C5—C2—C3—C7	−39.77 (15)	C24—N3—C15—O4	−3.7 (2)
C1—C2—C3—C7	83.61 (13)	C16—N3—C15—N2	0.86 (19)
C5—C2—C3—C4	−158.76 (11)	C24—N3—C15—N2	177.18 (12)
C1—C2—C3—C4	−35.38 (13)	C6—N2—C15—O4	175.59 (13)
C1—N1—C4—C25	114.32 (12)	C23—N2—C15—O4	2.7 (2)
S1—N1—C4—C25	−101.05 (11)	C6—N2—C15—N3	−5.27 (19)
C1—N1—C4—C3	−9.10 (14)	C23—N2—C15—N3	−178.21 (12)
S1—N1—C4—C3	135.53 (10)	C15—N3—C16—O5	−175.14 (12)
C7—C3—C4—N1	−90.39 (12)	C24—N3—C16—O5	8.55 (18)
C2—C3—C4—N1	27.02 (13)	C15—N3—C16—C7	5.83 (19)
C7—C3—C4—C25	148.58 (11)	C24—N3—C16—C7	−170.48 (12)
C2—C3—C4—C25	−94.01 (12)	C6—C7—C16—O5	172.74 (13)
C6—O3—C5—C17	78.79 (14)	C3—C7—C16—O5	−8.3 (2)
C6—O3—C5—C2	−46.30 (15)	C6—C7—C16—N3	−8.28 (18)
C1—C2—C5—O3	−58.54 (14)	C3—C7—C16—N3	170.68 (11)
C3—C2—C5—O3	59.67 (14)	O3—C5—C17—C22	14.05 (17)
C1—C2—C5—C17	177.71 (11)	C2—C5—C17—C22	137.41 (13)
C3—C2—C5—C17	−64.08 (14)	O3—C5—C17—C18	−167.34 (12)
C5—O3—C6—C7	14.77 (19)	C2—C5—C17—C18	−43.98 (17)
C5—O3—C6—N2	−165.52 (11)	C22—C17—C18—C19	−1.5 (2)
C15—N2—C6—O3	−177.06 (12)	C5—C17—C18—C19	179.87 (14)
C23—N2—C6—O3	−4.32 (18)	C17—C18—C19—C20	0.4 (2)
C15—N2—C6—C7	2.6 (2)	C18—C19—C20—C21	0.4 (3)
C23—N2—C6—C7	175.40 (13)	C19—C20—C21—C22	−0.1 (3)
O3—C6—C7—C16	−175.88 (12)	C20—C21—C22—C17	−1.0 (3)
N2—C6—C7—C16	4.4 (2)	C18—C17—C22—C21	1.8 (2)
O3—C6—C7—C3	5.2 (2)	C5—C17—C22—C21	−179.60 (15)
N2—C6—C7—C3	−174.49 (12)	N1—C4—C25—C26	−65.58 (15)
C2—C3—C7—C6	8.60 (17)	C3—C4—C25—C26	51.48 (16)
C4—C3—C7—C6	122.47 (13)	C4—C25—C26—C31	−98.15 (16)
C2—C3—C7—C16	−170.33 (11)	C4—C25—C26—C27	77.65 (17)
C4—C3—C7—C16	−56.46 (16)	C31—C26—C27—C28	2.7 (2)
O2—S1—C8—C13	159.31 (11)	C25—C26—C27—C28	−173.20 (14)
O1—S1—C8—C13	28.42 (13)	C26—C27—C28—C29	−1.6 (2)
N1—S1—C8—C13	−85.74 (12)	C27—C28—C29—C30	−0.7 (3)
O2—S1—C8—C9	−20.53 (13)	C28—C29—C30—C31	1.8 (3)
O1—S1—C8—C9	−151.41 (11)	C27—C26—C31—C30	−1.6 (2)
N1—S1—C8—C9	94.43 (12)	C25—C26—C31—C30	174.38 (14)
C13—C8—C9—C10	−1.9 (2)	C29—C30—C31—C26	−0.7 (2)

### Hydrogen-bond geometry (Å, °)

**Table:**

*D*—H···*A*	*D*—H	H···*A*	*D*···*A*	*D*—H···*A*
C4—H4···O5	0.98	2.45	3.0586 (16)	120
C2—H2···Cg1	0.98	2.67	3.5377 (15)	148
C22—H22···O4^i^	0.93	2.57	3.258 (2)	132
C24—H24B···O5^ii^	0.96	2.48	3.4236 (17)	169
C30—H30···O1^iii^	0.93	2.40	3.180 (2)	142
C25—H25A···Cg1^iv^	0.97	2.88	3.5110 (15)	123

Symmetry codes: (i) −*x*, −*y*+1, −*z*+1; (ii) −*x*, −*y*+1, −*z*; (iii) −*x*+1, *y*+1/2, −*z*+1/2; (iv) −*x*+1, −*y*+1, −*z*.
